# Obstetric and Neonatal Outcomes of Using Mechanical Methods in Cases of Pregnancy Requiring Cervical Ripening Following Premature Rupture of the Membranes at Term

**DOI:** 10.31662/jmaj.2024-0372

**Published:** 2025-09-12

**Authors:** Nobuko Yokoyama, Shunji Suzuki

**Affiliations:** 1 Department of Obstetrics and Gynecology, Japanese Red Cross Katsushika Maternity Hospital, Tokyo, Japan

**Keywords:** cervical ripening, mechanical methods, premature rupture of the membranes, term

## Abstract

**Introduction:**

We examined the obstetric and neonatal outcomes of using mechanical methods (i.e., insertion of Dilapan as the synthetic equivalent in the cervical canal) for cervical ripening in cases with premature rupture of the membranes (PROMs) at term.

**Methods:**

The criteria for inclusion in this retrospective observational study were as follows: PROM, singleton pregnancy, cephalic presentation, gestational age of 37-41 weeks, and a Bishop score of 0-2 at the beginning of induction. During the study period, 85 women met the criterion, and mechanical methods were used for cervical ripening following PROM. We examined the rate of cesarean delivery, number of days from the start of induction to delivery, umbilical artery pH, the incidence of clinical intrauterine infection, and neonatal infection.

**Results:**

In cases of using mechanical methods following PROM at term, the number of days from the start of induction to delivery was 2.1 ± 2 days. The rate of cesarean delivery was 26% (22/85). The incidence of clinical intrauterine infection during labor was 7% (6/85), while there were no cases of neonatal infection. In addition, the incidence of maternal fever after the third postoperative day was 1% (1/85).

**Conclusions:**

The current results suggest the potential clinical utility of mechanical cervical ripening methods following PROM at term.

## Introduction

In pregnant women requiring labor induction with an unfavorable cervix, cervical ripening is usually the first step. Recently, prostaglandin preparations (oral prostaglandin E2 or controlled-release dinoprostone vaginal delivery systems) have been used as methods for cervical ripening in Japan ^(1), (2), (3), (4), (5), (6), (7), (8)^; however, mechanical methods (i.e., insertion of laminaria tents or their synthetic equivalent such as Dilapan in the cervical canal) have also been options for the cervical ripening ^(3), (6)^. The research has indicated that both options are effective similarly, and combining them can be successful for cervical ripening in pregnant women with an unfavorable cervix without premature rupture of the membranes (PROMs) at term ^(9), (10), (11)^. However, in cases with PROM, compared with the use of pharmacologic agents alone, maternal and neonatal infectious morbidity appeared to be increased when mechanical methods are used for cervical ripening ^(12)^. In contrast, a very low Bishop score at the start of cervical ripening has been feared to indicate poor prostaglandin preparations’ efficacy, because in women with very low Bishop scores, mechanical methods have been suggested as superior or alternative treatments to prostaglandin preparations ^(5), (13), (14), (15)^. These previous observations usually concern us about the choice of cervical ripening methods when confronted with a pregnant woman with PROM who has a very low Bishop score of about 0-2.

Therefore, in this study, we examined the obstetric and neonatal outcomes of using mechanical methods for cervical ripening in cases with a low Bishop score with PROM at term.

## Materials and Methods

This retrospective observational study was conducted at our institute, one of the main perinatal centers in Tokyo, Japan, between 2022 and 2023. The current study was granted approval by the Ethics Committee of our institute: the Japanese Red Cross Katsushika Maternity Hospital (K24-12), and all participants gave informed consent before the implementation of cervical ripening. In our institute, we do not use the other largely used mechanical methods, such as single or double balloon, in cases with PROM due to the risk of umbilical cord prolapse. In our institute, in cases of PROM beyond 37 weeks of gestation, induction of labor is performed at 9:00 a.m. of the day following the day of the PROM if there are no findings of intrauterine infection suspected such as maternal fever of 38°C or higher, vaginal odor discharge, and/or tachycardia in either mother (>100 bpm) or fetus (>180 bpm) ^(12), (16)^. We have selected these methods to avoid induction in the middle of the night. In these cases, the use of contractions (oxytocin) or cesarean section was considered regardless of the degree of cervical ripening for the same-day delivery. Clinical data on maternal characteristics and obstetric outcomes were obtained from hospital records. In this study, the criteria for inclusion in this study were as follows: PROM, singleton pregnancy, cephalic presentation, gestational age of 37-41 weeks, and a Bishop score of 0-2 at the beginning of induction. In this study, we excluded the cases in which the onset of labor pains had occurred before the start of induction, the cases in which intrauterine infection was suspected with the aforementioned findings ^(12), (16)^, and the cases with a Bishop score of 3 or higher at the start of induction. In cases with a Bishop score of 3 or higher, prostaglandin preparations were usually used in our institute in anticipation of response to prostaglandins, with the assessment that mechanical methods have already outstripped the situation that needs to be addressed ^(5), (13), (14), (15)^. If there is any doubt about the diagnosis of intrauterine infection, blood tests are used to assess infection/inflammatory findings; however, if the mother or infant has fever and/or tachycardia, blood tests are sometimes omitted because the impact of fever and/or tachycardia on the full-term fetus is significant ^(16), (17)^. The cases with perinatal complications such as hypertensive disorders and oligohydramnios (i.e., amniotic fluid index <5 before PROM) were excluded. During the study period, 85 women met the criterion, and mechanical methods were used for cervical ripening following PROM. During the study period, we used Dilapan-S as a synthetic equivalent osmotic dilator in the cervical canal ^(6)^. For insertion of the Dilapan, a vaginal speculum was inserted, and the vaginal canal was washed with a saline solution. The cervix was then held with a single hook forceps, and 1 to 5 pieces of Dilapan-S, pinched with the forceps, were inserted into the cervix canal slowly to reach the internal ostium of the uterus, and held in place with a gauze moistened with saline solution. The number of Dilapan-S was increased as much as possible up to 5. If the procedure did not result in effective labor, prostaglandin preparations or oxytocin were used for additional cervical ripening or augmentation of labor.

We examined maternal age, parity, gestational age at PROM, maternal body mass index and Bishop score at the start of induction, oxytocin use, rate of cesarean delivery, number of days from the start of induction to delivery, neonatal birth weight, neonatal asphyxia (i.e., Apgar score at 1 minute <7), umbilical artery pH, and the incidence of 2 types of intrauterine infection as follows: one is a clinical manifestation of intrauterine infection during labor defined as maternal fever of 38°C or higher accompanied by fetal tachycardia (>180 bpm), and the other is signs of neonatal infection ^(11), (12), (18)^. Neonatal infection (i.e., fetal intrauterine infection) was defined as a clinical manifestation diagnosed by neonatologists based on the following criteria: respiratory distress, blood test findings, and chest X-ray findings. In addition, maternal fever of 38°C or higher after the third postoperative day was considered suggestive of maternal infection.

During this study period, 48 women with a Bishop score of 3 were induced labor without using mechanical methods following PROM. The 48 cases were additionally studied to examine the safety of our aforementioned cervical ripening method.

Data are presented as numbers (percentages) or mean ± standard deviation. Fortunately, there were no missing values in these clinical data. In addition, unpaired t-test or chi-square test were used to compare the results with the obstetric outcomes of the women with a Bishop score of 3 without using mechanical methods following PROM, and p < 0.05 was considered significant.

## Results

Table 1 shows the clinical characteristics of patients in this study, while Table 2 shows the obstetric outcomes in this study. On the day of induction start, 2 cases were spontaneously delivered vaginally. The following day, the average Bishop score of the remaining 83 cases was 3.6 ± 2. Prostaglandin preparations were used in 45 cases, and repeat mechanical methods were used in 12 cases for additional cervical ripening, while oxytocin was used in 26 cases to augment labor because of a Bishop score of 6 or more. Oxytocin was also used in the other 33 cases from the following day onward; as a result, oxytocin was used in a total of 59 cases (69%). The number of days from the start of induction to delivery was 2.1 ± 2 days. The rate of cesarean delivery was 26% (n = 22). Six cases (7%) were complicated by clinical intrauterine infection, as shown in Table 2; however, there were no cases of neonatal infection. In addition, the incidence of maternal fever after the third postoperative day was 1% (n = 1).

**Table 1.** Clinical Characteristics of Patients.

Total number	85
Maternal age (y)	33.7 ± 7
Nulliparity	74 (87)
Gestational age at PROM (w)	39.4 ± 2
Body mass index at the start of induction	24.9 ± 3.3
Bishop score at the start of induction	1.3 ± 1
Neonatal birth weight (g)	3170 ± 340

Data are presented as numbers (percentages) or mean ± standard deviation. PROM, premature rupture of the membranes.

**Table 2.** Obstetric Outcomes.

Total number	85
Findings and procedures on the second day of intervention	
After delivery	2 (2)
Prostaglandin preparations	45 (53)
Repeat mechanical methods	12 (14)
Oxytocin use	26 (31)
Total oxytocin use	59 (69)
Number of days from the start of induction to delivery (d)	2.1 ± 2
Total cesarean delivery	22 (26)
Indication for cesarean delivery (n = 22)	
Intrauterine infection	5 (23)
Non-reassuring fetal status	9 (41)
Arrest of labor	8 (36)
Apgar score at 1 minute	8.3 ± 1
Neonatal asphyxia (Apgar score at 1 minute <7)	0 (0)
Neonatal asphyxia (Apgar score at 5 minutes <7)	0 (0)
Umbilical artery pH	7.247 ± 0.10
Umbilical artery pH <7.1	1 (1)
Clinical intrauterine infection	6 (7)
Neonatal intrauterine infection	0 (0)
Maternal fever after the third postoperative day	1 (1)

Data are presented as numbers (percentages) or mean ± standard deviation.

Of the 48 women with a Bishop score of 3 without using mechanical methods following PROM, 15 cases (31%, p = 0.51) resulted in cesarean section, and 2 cases (4%, p = 0.50) were diagnosed as clinical intrauterine infection during labor. Table 3 shows the comparison with the current study cases. Although the comparison was performed under completely different conditions and methods, there did not appear to be any adverse effect of our selection of cervical ripening methods on the obstetric and neonatal outcomes, as shown in Table 3.

**Table 3.** Comparison of the Overview of the Cases of PROM with Mechanical Cervical Dilation and Those with the Pharmacological Ripening Methods.

Cervical ripening method	Mechanical methods	Pharmacological methods	P-value*
Number	85	48
Maternal age (y)	33.7 ± 7	32.0 ± 6	0.01
Nulliparity	74 (87)	34 (71)	0.02
Gestational age at PROM (w)	39.4 ± 2	39.0 ± 2	0.11
Neonatal birth weight (g)	3170 ± 340	3130 ± 330	0.01
Oxytocin use	59 (69)	21 (44)	<0.01
Number of days from the start of induction to delivery (d)	2.1 ± 2	1.8 ± 1	0.18
Total cesarean delivery	22 (26)	15 (31)	0.51
Indication for cesarean delivery (n = 26 and 15)
Intrauterine infection	5 (23)	1 (7)	0.19
Non-reassuring fetal status	9 (41)	8 (53)	0.46
Arrest of labor	8 (36)	6 (40)	0.82
Apgar score at 1 minute	8.3 ± 1	8.7 ± 1	0.09
Neonatal asphyxia (Apgar score at 1 minute <7)	0 (0)	0 (0)	1
Neonatal asphyxia (Apgar score at 5 minutes <7)	0 (0)	0 (0)	1
Umbilical artery pH	7.247 ± 0.10	7.285 ± 0.11	0.52
Umbilical artery pH <7.1	1 (1)	0 (0)	0.45
Clinical intrauterine infection	6 (7)	2 (4)	0.53
Neonatal intrauterine infection	0 (0)	0 (0)	1
Maternal fever after the third postoperative day	1 (1)	0 (0)	0.45

Data are presented as numbers (percentages) or mean ± standard deviation. PROM, premature rupture of the membranes. *By unpaired t-test or chi-square test.

## Discussion

In this study, the incidence of clinical intrauterine infection was 7%; however, there were no cases of neonatal infection. In addition, the clinical incidence of maternal fever was only 1%. In this study, we did not perform the definitive diagnosis of intrauterine infection (chorioamnionitis) by placental pathology; however, fetal infection or maternal fever was not observed with mechanical methods for cervical ripening in cases with PROM at term. Taking into account the results of an additional study with pharmacological ripening methods, our strategy with mechanical methods against PROM did not appear to significantly increase the risk of intrauterine infection. In addition, the cesarean section rate in this study may not be considered high based on the current status of obstetric care in Japan ^(19)^. Although the possibility that cervical manipulation can cause intrauterine infection cannot be completely ruled out, we believe that we have demonstrated that intrauterine infection does not occur at a high incidence if the procedure is performed with the risk in mind.

In an earlier study by Heinemann et al. ^(12)^, maternal and neonatal infectious morbidity appeared to be increased when mechanical agents were used for cervical ripening compared with the use of pharmacologic agents alone. Although no comparative examination under similar backgrounds with prostaglandin preparations was conducted in this study, we believe that we have demonstrated that at least the mechanical methods can also be used in cases requiring cervical ripening with a very low Bishop score with PROM. For example, we may be able to recommend giving preference to the mechanical methods in cases with PROM complicated by oligohydramnios associated with a high risk of fetal asphyxia, or patient conditions that would make the use of prostaglandin preparations hesitant, such as a history of asthma.

We understand that there are some serious limitations other than the small number of subjects in this study. First of all, we must raise the possibility that the current results are underestimated because of the descriptive study without a comparison group with similar backgrounds. Without the comparison group, there may be no ability to understand the contribution of the mechanical methods to complications. However, we believe that the current observation helps negate fears of the risk in the mechanical methods, which is comparable to the group induced labor without mechanical methods, with a better Bishop score. Second, considering that 10%-25% of deliveries at term are preceded by PROM ^(20)^, it must also be necessary to examine the various possible scenarios in a large study. In this study, because the time interval between the PROM and intervention was not constant, the pure usefulness of the mechanical methods might not be examined, since in some cases, spontaneous labor had occurred by the time of intervention. In Japan, it has been common practice to wait until the following day because spontaneous labor occurs within 24 hours in approximately 80% of cases with PROM at term ^(20)^. Third, we have selected the current methods―which do not necessarily wait 24 hours―to avoid induction at midnight, and we believe the current results will support the methods as acceptable. Fourth, a comparison with a control group that follows a natural course without any medical intervention in cases with PROM would be necessary; however, it would be ethically impossible to set up such a group. Alternatively, a comparison with a group using prostaglandin preparations will be considered necessary for the purpose of expanding the range of options available to pregnant women with PROM.

In the current study, mechanical cervical ripening did not statistically significantly increase the incidence of intrauterine infection during labor in cases of PROM at term. Although the current study may be small, the current results suggest the potential clinical utility of mechanical cervical ripening methods following PROM at term.

## Article Information

### Author Contributions

Nobuko Yokoyakma: data management, data analysis, and manuscript writing.

Shunji Suzuki: project development, data management, data analysis, and manuscript writing and editing.

### Conflicts of Interest

None

### Consent

The current study was granted approval by the Ethics Committee of the Japanese Red Cross Katsushika Maternity Hospital (K24-12), and all participants gave informed consent prior to their inclusion.

## References

[1] Brunnberg FJ. Application of prostaglandins in obstetrics and gynecology. Acta Biol Med Ger. 1978;37(5-6):917-21. 742314

[2] Singh EJ, Zuspan FP. Prostaglandins: overview in obstetrics and gynecology. J Reprod Med. 1974;12(5):211-4. 4599109

[3] ACOG practice bulletin no. 107: induction of labor. Obstet Gynecol. 2009;114(2 Pt 1):386-97. 19623003 10.1097/AOG.0b013e3181b48ef5

[4] Karim SM, Sharma SD. Oral administration of prostaglandins for the induction of labour. Br Med J. 1971;1(5743):260-2. 5542159 10.1136/bmj.1.5743.260 PMC1794953

[5] Itoh H, Ishii K, Shigeta N, et al. Efficacy and safety of controlled-release dinoprostone vaginal delivery system (PROPESS) in Japanese pregnant women requiring cervical ripening: results from a multicenter, randomized, double-blind, placebo-controlled phase III study. J Obstet Gynaecol Res. 2021;47(1):216-25. 33094550 10.1111/jog.14472 PMC7820955

[6] de Vaan MD, Ten Eikelder ML, Jozwiak M, et al. Mechanical methods for induction of labour. Cochrane Database Syst Rev. 2023;3(3):CD001233. 36996264 10.1002/14651858.CD001233.pub4 PMC10061553

[7] Tseng JY, Lin IC, Chang WH, et al. Using dinoprostone vaginal insert for induction of labor: A single institute experience. Taiwan J Obstet Gynecol. 2020;59(5):723-7. 32917325 10.1016/j.tjog.2020.07.017

[8] Nien YC, Kung HF, Chen MJ, et al. Dinoprostone tablet versus continuous vaginal insert (Propess)^Ⓡ^ for elective induction in low-risk nulliparous women at term. Taiwan J Obstet Gynecol. 2023;62(6):858-62. 38008505 10.1016/j.tjog.2023.03.016

[9] Ezebialu IU, Eke AC, Eleje GU, et al. Methods for assessing pre-induction cervical ripening. Cochrane Database Syst Rev. 2015;2015(6):CD010762. 26068943 10.1002/14651858.CD010762.pub2 PMC4473357

[10] Yamaguchi M, Takakura S, Enomoto N, et al. Comparison of perinatal outcomes between controlled-release dinoprostone vaginal delivery system (PROPESS) and metreurynter for cervical ripening in labor induction: a retrospective single-center study in Japan. J Obstet Gynaecol Res. 2021;47(12):4256-62. 34545652 10.1111/jog.15036

[11] Shibata Y, Yokoyama N, Suzuki S. A retrospective comparative study of the effect of controlled-release dinoprostone vaginal delivery System (Propess^Ⓡ^) and mechanical methods for cervical ripening in nulliparous women in late-term pregnancy. Cureus. 2023;15(10):e47255. 37859678 10.7759/cureus.47255 PMC10584270

[12] Heinemann J, Gillen G, Sanchez-Ramos L, et al. Do mechanical methods of cervical ripening increase infectious morbidity? A systematic review. Am J Obstet Gynecol. 2008;199(2):177-87; discussion 187. 18674661 10.1016/j.ajog.2008.05.005

[13] Imai K, Nozaki Y, Ushida T, et al. Comparison of the efficacy between controlled-release dinoprostone delivery system (PROPESS) and Cook’s double balloon catheter plus oxytocin: a retrospective single-center study in Japan. J Obstet Gynaecol Res. 2023;49(9):2317-23. 37385818 10.1111/jog.15734

[14] Shindo R, Aoki S, Nakanishi S, et al. Impact of introducing a controlled-release dinoprostone vaginal insert for labor induction: a retrospective single-center study in Japan. Cureus. 2024;16(1):e53180. 38420080 10.7759/cureus.53180 PMC10901581

[15] Takizawa A, Tsunoda Y, Matsushima T, et al. Factors associated with insufficient cervical ripening in a controlled-release dinoprostone vaginal delivery system: a single perinatal center retrospective study. Taiwan J Obstet Gynecol. 2024;63(6):887-91. 39481997 10.1016/j.tjog.2024.05.026

[16] Suzuki S. Association between clinical chorioamnionitis and histological funisitis at term. J Neonatal Perinatal Med. 2019;12(1):37-40. 30040750 10.3233/NPM-17155

[17] Suzuki S. Obstetric management during painless vaginal delivery (in Japanese). Acta Obstet Gynaecol Jpn. 2020;72(12):1764-7.

[18] Imai K, Aoki C, Tano S, et al. Clinical significance of bishop score and vaginal bleeding to controlled-release dinoprostone delivery system (PROPESS) efficacy for cervical ripening: a retrospective single-center study in Japan. J Obstet Gynaecol Res. 2023;49(4):1154-60. 36746752 10.1111/jog.15575

[19] Guidelines for establishing a system for perinatal care [Internet]. Ministry of Health, Labour, and Welfare. 2024 [cited 2024 Nov 17]. https://www.mhlw.go.jp/content/10800000/001118039.pdf. Japanese.

[20] Yoneda T. Premature rupture of membranes (term). Perinat Med (Tokyo). 2021;51:S321-3. Japanese.

